# Prediction of chronic kidney disease after orthotopic liver transplantation: development and validation of a nomogram model

**DOI:** 10.1186/s12882-021-02650-1

**Published:** 2022-01-16

**Authors:** Dandan Guo, Huifang Wang, Jun Liu, Hang Liu, Ming Zhang, Zixuan Fu, Xuemei Liu

**Affiliations:** Department of Nephrology, The Affiliated Hospital of Qingdao University, No. 16 Jiangsu Road, Qingdao, 266003 Shandong China

**Keywords:** Chronic kidney disease, Orthotopic liver transplantation, Nomogram model, Prognosis

## Abstract

**Background:**

We aimed to develop and validate a nomogram model for predicting CKD after orthotopic liver transplantation (OLT).

**Methods:**

The retrospective data of 399 patients who underwent transplantation and were followed in our centre were collected. They were randomly assigned to the training set (*n* = 293) and validation set (*n* = 106). Multivariable Cox regression analysis was performed in the training set to identify predictors of CKD. According to the Cox regression analysis results, a nomogram model was developed and validated. The renal function of recipients was monitored, and the long-term survival prognosis was assessed.

**Results:**

The incidence of CKD at 5 years after OLT was 25.6%. Cox regression analysis identified several predictors of post-OLT CKD, including recipient age at surgery (HR 1.036, 95% CI 1.006-1.068; *p* = 0.018), female sex (HR 2.867, 95% CI 1.709-4.810; *p* < 0.001), preoperative hypertension (HR 1.670, 95% CI 0.962-2.898; *p* = 0.068), preoperative eGFR (HR 0.996, 95% CI 0.991-1.001; *p* = 0.143), uric acid at 3 months (HR 1.002, 95% CI 1.001-1.004; *p* = 0.028), haemoglobin at 3 months (HR 0.970, 95% CI 0.956-0.983; *p* < 0.001), and average concentration of cyclosporine A at 3 months (HR 1.002, 95% CI 1.001-1.003; *p* < 0.001). According to these parameters, a nomogram model for predicting CKD after OLT was constructed and validated. The C-indices were 0.75 and 0.80 in the training and validation sets. The calibration curve of the nomogram showed that the CKD probabilities predicted by the nomogram agreed with the observed probabilities at 1, 3, and 5 years after OLT (*p* > 0.05). Renal function declined slowly year by year, and there were significant differences between patients divided by these predictors. Kaplan-Meier survival analysis showed that the survival prognosis of recipients decreased significantly with the progression of renal function.

**Conclusions:**

With excellent predictive abilities, the nomogram may be a simple and reliable tool to identify patients at high risk for CKD and poor long-term prognosis after OLT.

## Introduction

Chronic kidney disease (CKD) is a common and significant complication after orthotopic liver transplantation (OLT). With the progression of surgical technology, perioperative treatment, and immunosuppressants, the survival rates of patients after OLT have dramatically improved to 90 and 75% at 1 and 5 years after OLT, respectively [1]. Long-term complications, such as CKD and cardiovascular events, are receiving increasing attention from clinical specialists. The prevalence of CKD reported in the literature was 4 ~ 27.5% in 1 year [2–4], 15 ~ 60% in 5 years [2, 4, 5], and 25 ~ 50% in 10 years after OLT [6, 7]. CKD is a primary contributor to morbidity and mortality after OLT, including an increased risk of allograft dysfunction, cardiovascular events [8], and death [4, 9], which has elicited concern regarding post-OLT management. However, only a limited number of studies have assessed long-term renal outcomes after OLT, which has important implications for patient management.

Studies have shown that the causes of CKD after OLT are complex and may include preoperative (age, female sex, history of diabetes mellitus (DM) or hypertension, hyperlipidaemia, and hepatitis C) and postoperative (calcineurin inhibitor (CNI) toxicity, prolonged ischaemia, and haemodynamic instability) factors [6, 10–13]. There have been controversial results regarding a direct association between perioperative AKI and post-OLT CKD [14, 15].

A credible and helpful predictive model to identify which patients are at high risk of developing CKD in clinical practice has not been developed, although it would be a useful tool for designing preventative measures, such as early decreased doses or withdrawal of CNIs and administration of non-nephrotoxic immunosuppressants. In this study, we aimed to develop and validate a reliable nomogram model for predicting CKD after OLT. With this model, we can identify patients at high risk for CKD early and take targeted measures to prevent or slow the development and progression of CKD.

## Methods

### Study population

**Fig. 1:**
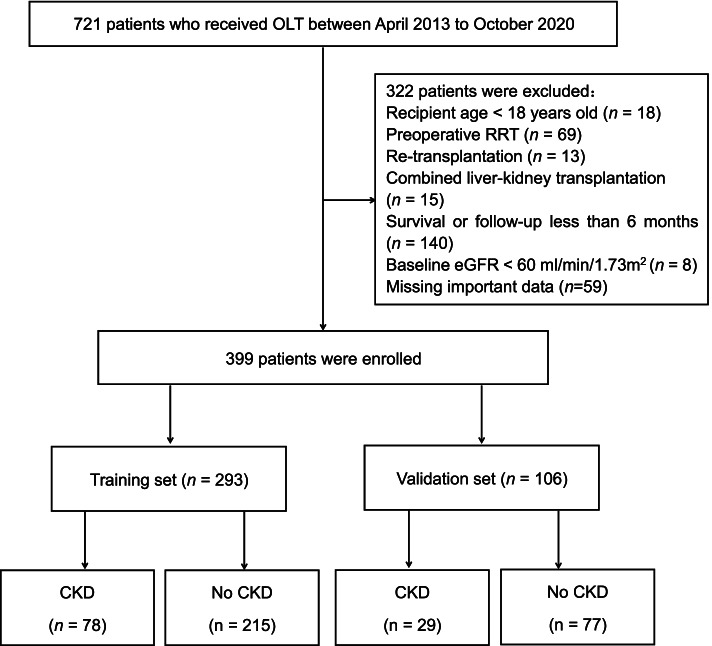
Flow diagram of patient enrolment

All adult patients who underwent OLT from April 2013 to October 2020 at our centre were enrolled in our study, and their medical records were retrospectively reviewed. Recipients with a minimal follow-up time of 6 months after transplantation were included. The exclusion criteria included retransplantation, baseline estimated glomerular filtration rate (eGFR) < 60 ml/min/1.73 m^2^, renal replacement therapy (RRT) before liver transplantation, multiorgan transplantation, follow-up less than 6 months, and age < 18 years. Finally, 399 patients were enrolled in our study. They were randomly allocated to the training set (*n* = 293) and the validation set (*n* = 106) (Fig. 1).

The study was conducted according to the principles of the Declaration of Helsinki and approved by the Medical Ethics Committee of The Affiliated Hospital of Qingdao University (the ethics approval number is QYFY WZLL 26283).

### Data collection and definitions

Demographic details, relevant preoperative and postoperative laboratory test results, and concentrations of CNIs were recorded. AKI was defined as an increase in serum creatinine (sCr) of at least 26.5 μmol/l within 48 h or more than 50% within 7 days after transplantation. Peak aspartate aminotransferase (AST) within the first 24 h after transplantation was used as a measure of ischaemia-reperfusion injury (IRI) [16]. New-onset CKD was defined as CKD that occurs after OLT and as eGFR < 60 ml/min/1.73 m^2^ for 3 months, regardless of the presence or absence of structural kidney damage [9]. sCr, eGFR, and CNI levels were recorded before OLT as well as 1-7 days and 1, 3, 6, and 12 months after OLT and yearly thereafter until the end of follow-up. eGFR was calculated according to the Modification of Diet in Renal Disease (MDRD) equation: eGFR = 186 × creatinine (mg/dL)^− 1.154^ × (age)^− 0.203^ × 1.212 (if black) × 0.742 (if female) [17].

According to the Kidney Disease: Improving Global Outcomes (KDIGO) CKD Work Group, the patients’ renal function was classified as follows [18]: mildly decreased: GFR 60-89 ml/min/1.73 m^2^; mildly to severely decreased: GFR 30-59 ml/min/1.73 m^2^, severely decreased: GFR 15-29 ml/min/1.73 m^2^; and kidney failure: GFR < 15 ml/min/1.73 m^2^ [19].

### Immunosuppression

The standard immunosuppressive regimen after OLT consisted of CNIs combined with mycophenolate mofetil (MMF) and corticosteroids. Most patients were treated with a triple-drug regimen of tacrolimus (FK506) or cyclosporine A (CsA), MMF, and corticosteroids. Methylprednisolone was given on the day of transplantation, followed by tapering, and then converted to methylprednisolone for oral administration, which was gradually reduced. When renal function was impaired, the introduction of CNIs was delayed. Some patients were switched to mammalian target of rapamycin (mTOR) inhibitor therapy when plasma FK506 failed to reach target levels or if CKD developed. The dose of immunosuppressant was adjusted according to blood concentration.

### Statistical analysis

R version 4.0.3 (R Foundation for Statistical Computing), STATA 15.0 (StataCorp, Texas, USA), and SPSS 25.0 were used to analyse the data. The median and interquartile range (IQR) were used to describe continuous variables, and the Mann–Whitney *U* test was performed to compare parametric variables. Categorical variables were expressed as quantities and percentages, and comparisons were made using the chi-square test or Fisher’s exact test. A Cox proportional hazards model was implemented in the training set to identify predictors of the development of CKD after liver transplantation. Factors with *p* < 0.05 in the univariable Cox analysis and several factors with clinical significance in previous studies were enrolled in the multivariable Cox analysis. The final Cox regression model was chosen by using forward stepwise regression with the Akaike information criterion (AIC) as the stopping rule. The AIC value for the final model was minimized with the fewest number of variables [20]. For further analysis, a nomogram was constructed based on the results of the Cox regression analysis. Then, we validated the predictive abilities of this model by examining its discrimination and calibration in the training set and validation set. The discrimination was quantified by the C-index, and the calibration was evaluated by the calibration curve. Kaplan–Meier curve analysis with the log-rank test was performed to evaluate the survival time of patients with renal function in different categories. The “rms” package was used for the nomogram and calibration curve. The “survminer” and “survival” packages were used for Kaplan–Meier analysis. For all statistical analyses, *p*-values of < 0.05 were considered statistically significant.

## Results

### Characteristics of the population

A total of 399 patients were enrolled in our study with a median follow-up of 31.5 months (IQR 18.3-51.6 months), of whom 293 patients were allocated to the training set and 106 patients were allocated to the validation set. The median age at transplantation of all patients was 52 years (IQR 45-59 years), and the majority of recipients were males (82.9%). The main indications for OLT at our centre were hepatitis B virus (HBV)-associated hepatocellular carcinoma (49.1%), followed by HBV-associated cirrhosis (27.5%). During the first week after liver transplantation, 71.1% of the recipients developed AKI, and 36.8% developed severe AKI (stage 2-3).

### Incidence of CKD after OLT

During the first week after OLT, 44.4% of recipients experienced a significant decrease in eGFR of more than 50%, including 8.3% of patients who had an eGFR of less than 30 ml/min/1.73 m^2^. At 1 year, 13.0% of recipients had eGFR < 60 ml/min/1.73 m^2^, and 0.8% had eGFR < 30 ml/min/1.73 m^2^. The incidences of CKD at 1, 3, and 5 years after OLT were 11.0, 20.3, and 25.6%, respectively. During the entire follow-up period, 28.6% of the recipients had renal function decreaesd, and the majority (55.1%) had eGFR mildly decreased. The mildly to severely decreased eGFR, severely decreased eGFR and end-stage renal disease (ESRD) (eGFR < 15 ml/min/1.73 m^2^ or long-term RRT) were observed in 26.8, 1.5, and 0.5% of the recipients, respectively. Only one recipient received a kidney transplant 19 months after OLT at our centre.

### Cox regression analysis of predictors associated with the development of CKD after OLT

The incidences of CKD were 26.6 and 27.3% in the training set and validation set, respectively (*p* > 0.05). Except for several factors with *p* < 0.05, including sCr, eGFR before OLT, serum albumin (ALB), and platelet-to-lymphocyte ratio (PLR) at 3 months after OLT, there were no significant differences between the training set and validation set regarding the demographic details, relevant preoperative and postoperative laboratory test results, and concentrations of CNIs (*p* > 0.05) (Tables 1, 2, and 3). The factors with *p* < 0.05 in the univariable Cox analysis and several factors with clinical significance in previous studies were included in the multivariable Cox regression model. The multivariable Cox regression analysis identified several recipient predictors associated with post-OLT CKD, including recipient age at surgery (hazard ratio [HR] 1.036, 95% confidence interval [CI] 1.006-1.068; *p* = 0.018), female sex (HR 2.867, 95% CI 1.709-4.810; *p* < 0.001), preoperative hypertension (HR 1.670, 95% CI 0.962-2.898; *p* = 0.068), preoperative eGFR (HR 0.996, 95% CI 0.991-1.001; *p* = 0.143), uric acid (UA) at 3 months (HR 1.002, 95% CI 1.001-1.004; *p* = 0.028), haemoglobin (Hb) at 3 months (HR 0.970, 95% CI 0.956-0.983; *p* < 0.001), and average concentration of CsA at 3 months after OLT (HR 1.002, 95% CI 1.001-1.003; *p* < 0.001) (Table 4). Postoperative AKI was associated with the development of CKD only in the univariable Cox regression analysis. The impact of FK506 was also investigated, and its median levels at 1 and 3 months after OLT were evaluated. Interestingly, these levels were lower in patients who developed CKD (at 1 month CKD 6.6, IQR 4.9-8.5 μg/L; no CKD 8.3, IQR 6.4-10.6 μg/L; *p* < 0.001; at 3 months CKD 6.3, IQR 3.5-7.8 μg/L; no CKD 6.6, IQR 4.8-8.4 μg/L; *p* = 0.084).

**Table 1:** Perioperative characteristics of patients between the training set and validation set

Characteristic	Training set (*n* = 293)	Validation set (*n* = 106)	*p*-value
**Demographic data**			
Age (years)	52 (46-59)	51 (44-58)	0.27
Gender (N,%)			0.65
Female	52 (17.7%)	16 (15.1%)	
Male	241 (82.3%)	90 (84.9%)	
BMI (Kg/m^2^)	24.22 (22.32-26.40)	23.69 (21.11-26.61)	0.40
MAP (mmHg)	92 (85-98)	91 (84-97)	0.96
Personal history			
Smoking (N,%)	125 (42.7%)	53 (50.0%)	0.21
Alcoholism (N,%)	140 (47.8%)	52 (49.1%)	0.82
**Pathogenesis of liver disease**			
HBV hepatitis (N,%)	229 (78.2%)	82 (77.4%)	0.89
Alcoholic liver cirrhosis (N,%)	21 (7.2%)	8 (7.5%)	1.00
Hepatocellular carcinoma (N,%)	139 (47.4%)	57 (53.8%)	0.31
Cholestatic disease (N,%)	19 (6.5%)	4 (3.8%)	0.47
Liver complications			
Encephalopathy (N,%)	44 (15.0%)	11 (10.4%)	0.26
Ascites> 1 L (N,%)	25 (23.6%)	71 (24.2%)	1.00
**Baseline medical status**			
Diabetes mellitus (N,%)	55 (18.8%)	21 (19.8%)	0.89
Hypertension (N,%)	38 (13.0%)	12 (11.3%)	0.73
Coronary heart disease (N,%)	8 (2.7%)	5 (4.7%)	0.34
Abdominal surgery history (N,%)	29 (27.4%)	62 (21.2%)	0.22
Preoperative LVEF (%)	63 (61, 65)	62 (60, 64)	0.10
Preoperative nonselective β receptor blockers (N,%)	24 (8.2%)	7 (6.6%)	0.68
Preoperative diuretics (N,%)	110 (37.5%)	30 (28.3%)	0.10
**Preoperative scores**			
MELD score	12.94 (9.50-18.87)	12.59 (9.50-17.00)	0.31
Child-Pugh score	10 (8-14)	9 (8-11)	0.14
**Preoperative parameters**			
White blood cell (× 10^9^/L)	3.39 (2.28-4.91)	3.61 (2.26-5.33)	0.53
Neutrophils (× 10^9^/L)	2.28 (1.46-3.51)	2.43 (1.58-3.62)	0.57
Lymphocyte (× 10^9^/L)	0.67 (0.40-1.03)	0.65 (0.47, 1.11)	0.44
Red blood cell (× 10^12^/L)	2.97 (2.48-3.56)	3.01 (2.56-3.66)	0.57
Hemoglobin (g/L)	91 (76-111)	92 (78-112)	0.63
Platelet (× 10^9^/L)	65 (40-105)	67 (41-109)	0.94
Total protein (g/L)	59.00 (55.00-62.93)	59.62 (54.21-64.80)	0.56
Albumin (g/L)	35.32 (32.57-39.40)	36.20 (32.20-39.02)	0.97
TBIL (μmol/L)	35.91 (21.70-105.80)	37.27 (20.66-77.07)	0.33
DBIL (μmol/L)	16.00 (9.29-51.61)	14.40 (9.03-32.81)	0.40
IBIL (μmol/L)	18.88 (11.80-52.62)	18.54 (11.10-38.70)	0.31
ALT (U/L)	26.00 (17.00-49.00)	27.50 (18.00-53.00)	0.39
AST (U/L)	38.00 (25.00-66.00)	36.50 (26.00-68.00)	0.76
LDH (U/L)	159.00 (133.00-193.00)	158.00 (139.00-202.00)	0.53
BUN (mmol/L)	4.36 (3.56-5.44)	4.22 (3.41-5.73)	0.45
sCr (μmol/L)	60.00 (48.00-74.00)	65.00 (54.00-78.00)	0.01
UA (μmol/L)	231.00 (179.00-293.00)	247.00 (176.00-314.00)	0.29
eGFR (mL/min/1.73 m^2^)	126.44 (99.21-160.67)	111.75 (94.99, 142.60)	0.02
**Postoperative parameters**			
Peak AST (U/L)	1239 (636-2325)	991 (679-1774)	0.27
Postoperative RRT (N,%)	5 (4.7%)	16 (5.5%)	1.00
Postoperative hospital stay (day)	24 (20, 29)	25 (21, 32)	0.12
Length of ICU stay (day)	4 (3-5)	4 (3-5)	0.59
AKI	71 (67.0%)	213 (72.7%)	0.26
CKD	78 (26.6%)	29 (27.4%)	0.90

Continuous variables are displayed as median and interquartile ranges

*Abbreviations*: *BMI* Body mass index, *MAP* Mean arterial pressure, *LVEF* Left ventricular ejection fraction, *MELD* Model for End-Stage Liver Disease, *TBIL* Serum total bilirubin, *DBIL* Serum direct bilirubin, *IBIL* Serum indirect bilirubin, *ALT* Alanine aminotransferase, *AST* Aspartate aminotransferase, *LDH* Lactate dehydrogenase, *BUN* Blood urea nitrogen, *sCr* Serum creatinine, *UA* Uric acid, *eGFR* Estimated glomerular filtration rate, *Peak AST* AST peak value within first 24 h after OLT

**Table 2:** Characteristics of patients at 1-month after OLT between the training set and validation set

Characteristic	Training set (*n* = 293)	Validation set (*n* = 106)	*p*-value
White blood cell (× 10^9^/L)	5.23 (4.06-6.51)	4.55 (3.51-6.06)	0.02
Neutrophils (× 10^9^/L)	3.40 (2.42-4.64)	2.96 (2.13-4.09)	0.02
Lymphocyte (× 10^9^/L)	0.97 (0.63-1.39)	0.93 (0.65-1.29)	0.93
Hemoglobin (g/L)	114 (102-126)	113 (99-125)	0.70
Platelet (× 10^9^/L)	167 (109-253)	156 (103-232)	0.41
NLR	3.33 (2.29-5.68)	3.13 (2.21-4.41)	0.12
PLR	150.99 (111.34-252.34)	165.71 (106.17-238.22)	0.78
N/LP	0.021 (0.011-0.045)	0.020 (0.012-0.034)	0.38
Total protein (g/L)	65.68 (59.52-70.14)	65.16 (60.40-70.01)	0.81
Albumin (g/L)	40.58 (38.46-43.71)	40.60 (37.49-44.08)	0.73
TBIL (μmol/L)	17.52 (14.07-28.07)	19.81 (14.88-30.00)	0.12
DBIL (μmol/L)	8.32 (5.84-13.66)	9.50 (6.50-15.50)	0.12
IBIL (μmol/L)	9.30 (7.16-13.40)	10.40 (7.71-14.74)	0.19
ALT (U/L)	28.00 (16.00-53.00)	27.00 (15.00-56.00)	0.60
AST (U/L)	19.00 (14.00-30.00)	18.00 (13.00-26.00)	0.22
LDH (U/L)	167.00 (148.00-204.00)	167.00 (141.00-205.00)	0.69
Glu (mmol/L)	5.55 (4.80-6.82)	5.38 (4.69-6.75)	0.57
CysC (mg/L)	1.17 (1.01-1.47)	1.18 (1.01-1.39)	0.66
C1q (mg/L)	165 (139-198)	171 (142-199)	0.46
BUN (mmol/L)	6.17 (4.85-7.62)	5.82 (4.74-7.51)	0.43
sCr (μmol/L)	76.5 (56.0-92.0)	72.0 (54.0, 86.0)	0.30
UA (μmol/L)	323.00 (245.00-405.00)	307.00 (239.00-374.00)	0.19
eGFR (mL/min/1.73 m^2^)	95.48 (78.01-134.82)	104.88 (81.69-141.05)	0.36
Average concentration of FK506	7.90 (6.00-12.40)	7.80 (5.86-10.03)	0.76
Average concentration of CsA	707.55 (242.98-1219.70)	369.95 (261.80-949.25)	0.43
Average concentration of Rapamycin	2.75 (2.72-427.35)	1.68 (1.66-3.74)	0.40

Continuous variables are displayed as median and interquartile ranges

*Abbreviations*: *NLR* Neutrophil lymphocyte ratio, *PLR* Platelet lymphocyte ratio, *N/LP* Ratio of Neutrophil to lymphocyte and platelet, *TBIL* Serum total bilirubin, *DBIL* Serum direct bilirubin, *IBIL* Serum indirect bilirubin, *ALT* Alanine aminotransferase, *AST* Aspartate aminotransferase, *LDH* Lactate dehydrogenase, *Glu* Glucose, *CysC* Cystatin, *C1q* Complement C1q, *BUN* Blood urea nitrogen, *sCr* Serum creatinine, *UA* Uric acid, *eGFR* Estimated glomerular filtration rate, *FK506* Tacrolimus, *CsA* Cyclosporin A

**Table 3:** Characteristics of patients at 3-months after OLT between the training set and validation set

Characteristic	Training set (*n* = 293)	Validation set (*n* = 106)	*p*-value
Neutrophils (× 10^9^/L)	2.49 (1.86-3.50)	2.61 (1.92-3.57)	0.43
Lymphocyte (× 10^9^/L)	1.15 (0.88-1.54)	1.16 (0.8-1.57)	0.70
Hemoglobin (g/L)	130 (117-139)	130 (119-141)	0.64
Platelet (× 10^9^/L)	114 (90-175)	132 (93-186)	0.11
NLR	2.17 (1.49-3.06)	2.33 (1.58-3.50)	0.22
PLR	102.99 (76.47-151.76)	120.41 (87.33-161.27)	0.04
N/LP	0.018 (0.012-0.027)	0.018 (0.01-0.029)	0.92
Albumin (g/L)	41.60 (37.41-44.70)	42.80 (39.50-45.42)	0.02
TBIL (μmol/L)	15.26 (11.33-19.49)	15.30 (11.08-21.25)	0.88
DBIL (μmol/L)	5.22 (4.14-7.40)	5.48 (3.80-7.79)	0.89
ALT (U/L)	21.00 (15.00-37.00)	22.00 (14.00-36.00)	0.95
AST (U/L)	20.00 (16.00-27.00)	20.00 (16.00-30.00)	0.34
Glu (mmol/L)	5.47 (4.79-6.28)	5.37 (4.86-6.35)	0.87
CysC (mg/L)	1.135 (0.965-1.375)	1.125 (0.985-1.325)	0.99
C1q (mg/L)	168 (139-198)	171 (145-194)	0.74
BUN (mmol/L)	5.90 (4.91-7.58)	5.97 (4.94-6.97)	0.70
sCr (μmol/L)	85.00 (66.00-93.00)	80.00 (60.00-93.00)	0.19
UA (μmol/L)	359.00 (305.00-439.00)	356.00 (292.00-416.00)	0.38
eGFR (mL/min/1.73 m^2^)	92.76 (80.28-131.31)	99.72 (80.29-147.29)	0.24
Average concentration of FK506	7.00 (5.40-8.50)	7.35 (5.15-8.98)	0.51
Average concentration of CsA	390.60 (246.25-575.35)	121.28 (73.68-478.19)	0.09
Average concentration of Rapamycin	4.14 (3.59-5.12)	3.97 (2.92-4.51)	0.33

Continuous variables are displayed as median and interquartile ranges

*Abbreviations*: *NLR* Neutrophil lymphocyte ratio, *PLR* Platelet lymphocyte ratio, *N/LP* Ratio of Neutrophil to lymphocyte and platelet, *TBIL* Serum total bilirubin, *DBIL* Serum direct bilirubin, *IBIL* Serum indirect bilirubin, *ALT* Alanine aminotransferase, *AST* Aspartate aminotransferase, *LDH* Lactate dehydrogenase, *Glu* Glucose, *CysC* Cystatin, *C1q* Complement C1q, *BUN* Blood urea nitrogen, *sCr* Serum creatinine, *UA* Uric acid, *eGFR* Estimated glomerular filtration rate, *FK506* Tacrolimus, *CsA* Cyclosporin A

**Table 4:** Multivariable cox regression analysis for predictors of CKD

Factors	***b***	***HR***	95% CI	***P***-value
Age	0.035	1.036	1.006-1.068	0.018
Female	1.029	2.867	1.709-4.810	< 0.001
Hypertension	0.479	1.670	0.962-2.898	0.068
eGFR before surgery	−0.004	0.996	0.991-1.001	0.143
Uric acid at 3 months	0.002	1.002	1.001-1.004	0.028
Hemoglobin at 3 months	−0.031	0.970	0.956-0.983	< 0.001
Average concentration of CsA3 at 3 months	0.002	1.002	1.001-1.003	< 0.001

*Abbreviations*: *b* Coefficient, *HR* Hazard ratio, *CI* Confidence interval, *eGFR* Estimated glomerular filtration rate, *CsA* Cyclosporin A

### Development and validation of a nomogram model for predicting CKD after OLT

**Fig. 2:**
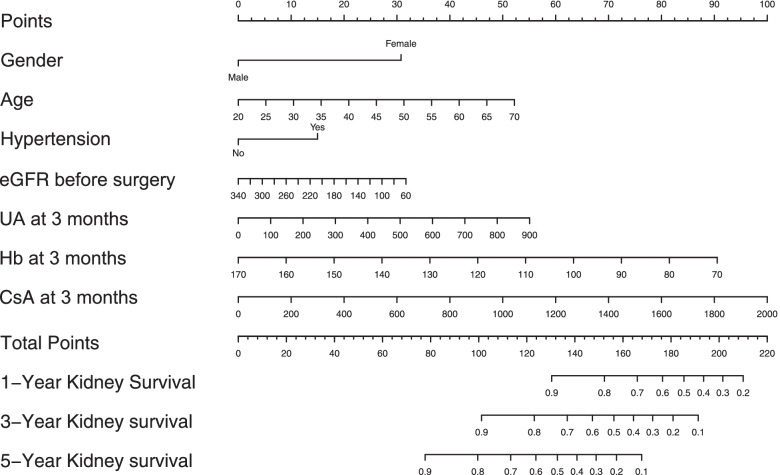
Nomogram for the prediction of CKD after OLT. Draw a vertical line towards the “Points” axis to determine the points of each variable, add the points and position the sum on the “Total Points” axis. Draw vertical lines towards the “1-Year kidney survival”, “3-Year kidney survival”, and “5-Year kidney survival” axes to find the probabilities of CKD at 1, 3, 5 years after OLT

**Fig. 3:**
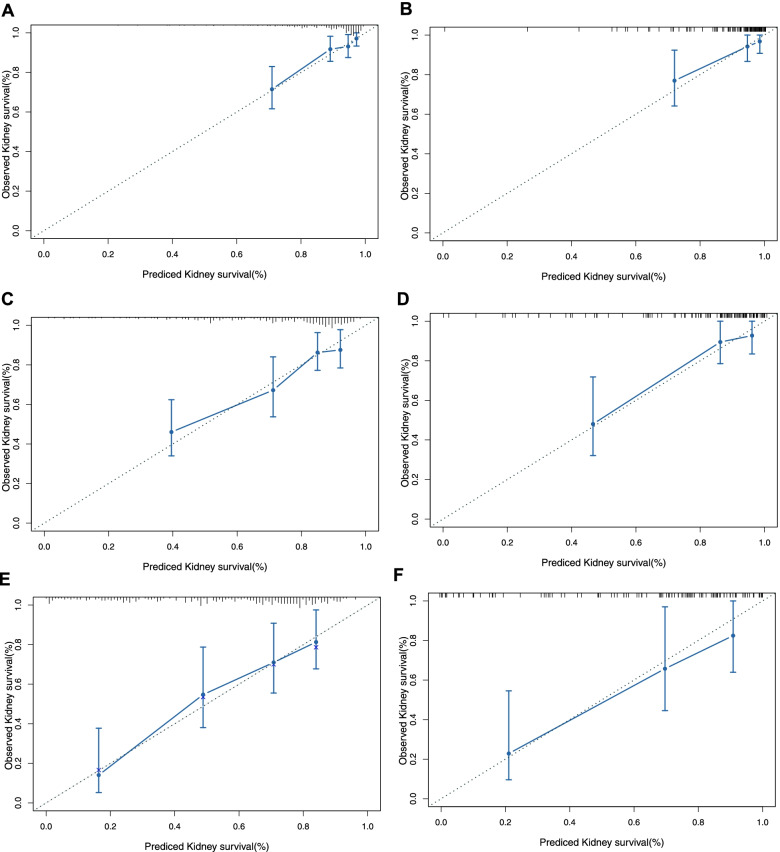
Calibration plot of the probabilities of CKD predicted by the nomogram model vs. the observed probabilities. Calibration at 1 year in the training set (**A**) and validation set (**B**). Calibration at 3 years in the training set (**C**) and validation set (**D**). Calibration at 5 years in the training set (**E**) and validation set (**F**) (all *p* > 0.05)

A nomogram model for predicting CKD after OLT was constructed based on the results of the multivariable Cox regression model (Fig. 2**)**. Points were assigned to the seven identified predictors based on their regression coefficients. For an individual patient, the points of each factor can be summed to calculate the estimated probabilities of CKD at 1, 3, and 5 years after OLT. Then, the nomogram was validated in the training set and validation set. The C-indices were 0.75 (95% CI 0.72-0.78) and 0.80 (95% CI 0.75-0.85) in the training set and validation set, respectively. The calibration curve of the nomogram showed that the CKD probabilities predicted by the nomogram agreed with the observed probabilities at 1, 3, and 5 years after OLT (*p* > 0.05) (Fig. 3). These results indicated that the nomogram model could accurately predict the risk of CKD after OLT using the perioperative predictors mentioned above.

### Renal recovery and long-term prognosis of recipients

**Fig. 4:**
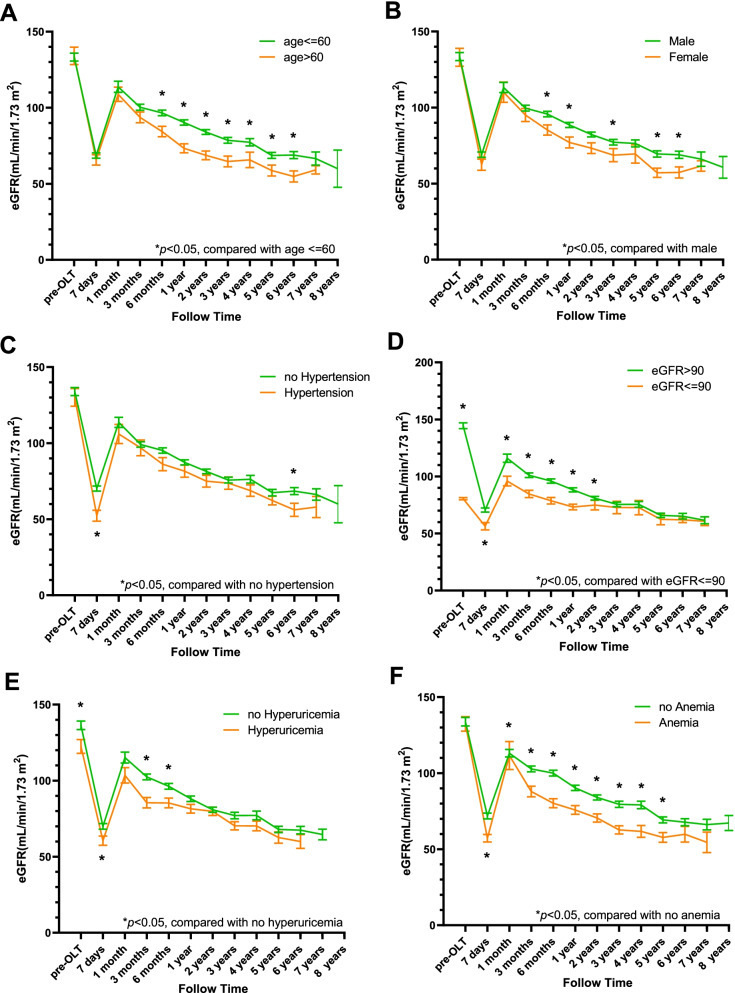
Time-dependent changes in renal function according to the predictors in the multivariable Cox regression analysis. *p < 0.05, compared with the reference group (nonparametric test)

**Fig. 5:**
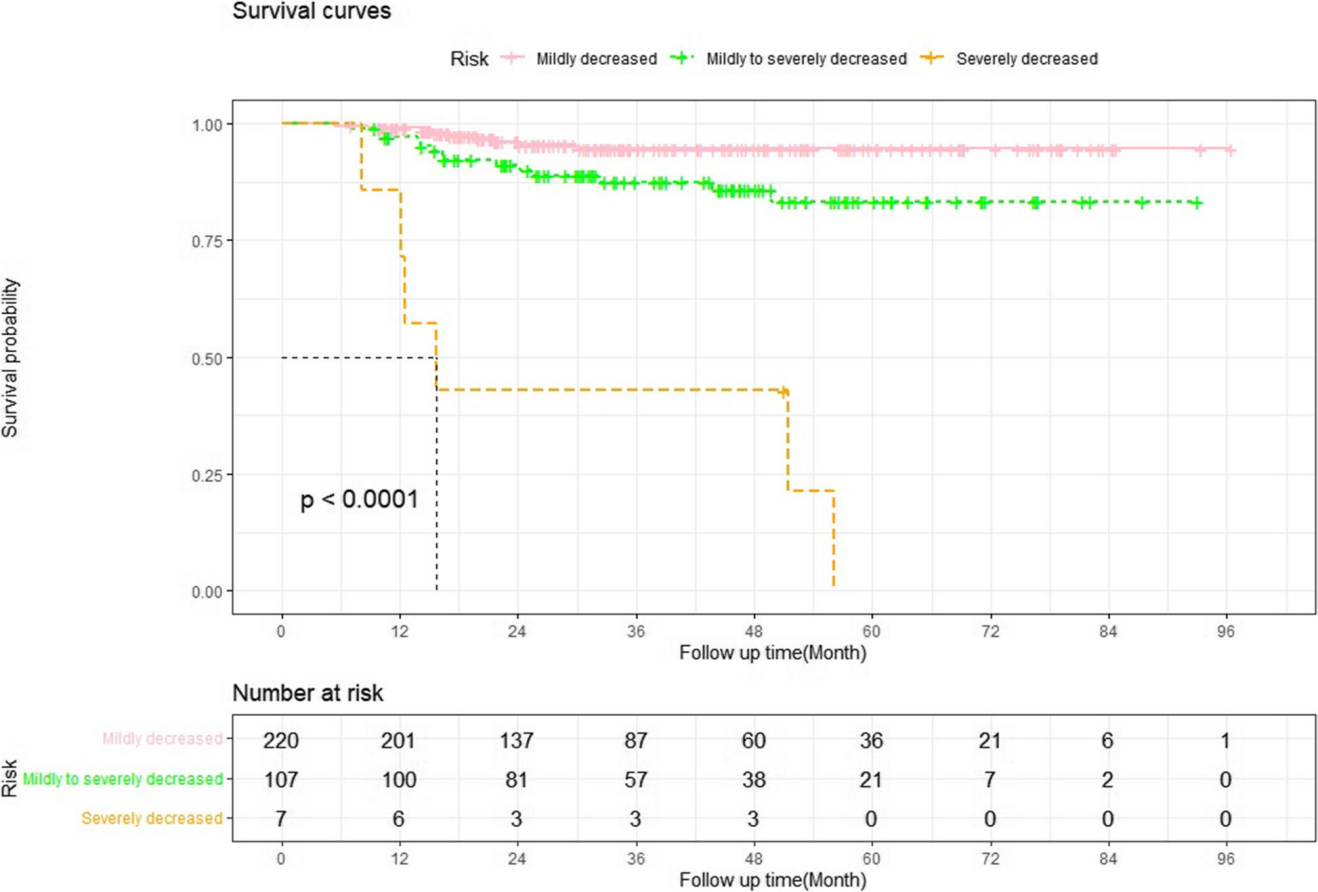
Patient survival compared between different renal function groups at the end of follow-up. mildly decreased: eGFR 60-89 mL/min/1.73 m^2^; mildly to severely decreased: eGFR 30-59 mL/min/1.73 m^2^; and severely decreased: eGFR 15-29 mL/min/1.73 m^2^ and ESRD

After the first week, renal function recovered rapidly in most recipients but decreased slowly after surgery. Based on the predictors of CKD in the multivariable analysis, including recipients’ age, sex, hypertension, preoperative eGFR, uric acid, and haemoglobin at 3 months after OLT, we divided the recipients into different groups. The differences in the changes in renal function between these groups are presented in Fig. 4. eGFR dropped sharply in the first 7 days after OLT due to postoperative AKI. Subsequently, eGFR increased sharply as renal function recovered and gradually decreased year by year. Because there were only a few patients with ESRD, they were included in the severely decreased renal function group. After a median follow-up period of 31.5 months (IQR 18.3-51.6 months), the mortality rates were 2.2, 4.1, and 1.8% in the mildly, mildly to severely, and severely decreased renal function groups, respectively (*p* < 0.001). Kaplan-Meier survival analysis with the log-rank test showed that survival was significantly different among these three groups (log-rank =70.829, *p* < 0.01, Fig. 5).

## Discussion

The increase in the number of long-term survivors of OLT raises questions about the increasing number of patients who develop CKD and ESRD. In our study, 25.6% of 399 recipients developed CKD at 5 years after OLT, which was consistent with that in previous reports as described earlier in this article. The survival curve showed that post-OLT CKD was a strong predictor of patient prognosis, including mortality. The time-dependent changes in renal function indicated that eGFR declined slowly year by year. It is important to identify patients at risk for CKD, especially in the early course, to slow the occurrence and progression of CKD.

Although different studies have attempted to identify the best predictors of renal dysfunction after liver transplantation, a reliable and simple model to accurately predict CKD is still lacking. For this reason, we chose to construct and validate a monogram model based on Cox regression analysis. A nomogram model can integrate multiple factors, enabling clinicians to comprehensively predict the prognosis of a patient and make clinical decisions. The model developed in our study integrated patients’ age at surgery, female sex, preoperative hypertension, baseline eGFR, UA, Hb, and average plasma concentration of CsA at 3 months after OLT as predictors of the development of CKD.

The first predictor in our model was female sex, which has previously been recognized as a risk factor for CKD. Female patients suffered a faster decline in renal function, as illustrated by the plot of the decrease in eGFR between males and females in this study. In contrast, among nontransplant patients, male sex was associated with a faster decline in eGFR. Female sex is also a risk factor for CKD after heart, lung, and kidney transplantation. The exact mechanism of this association is not clear. A possible explanation may be that females may be more susceptible to CNI-mediated renal injury [21].

Recipients with hypertension and low eGFR are more prone to suffering IRI during and after surgery due to trauma and circulation instability [6]. Although their *p* values were > 0.05, our nomogram model showed that preoperative hypertension and low eGFR were important predictors of new-onset CKD after OLT.

In our population study, the UA level at 3 months after OLT was found to be a risk factor for new-onset CKD. Elevated serum levels of UA are associated with the onset and progression of CKD in many populations, such as the general population [22], patients with hypertension [23], and kidney transplantation patients [24]. Although hyperuricaemia is common after OLT, its association with the development of CKD has not been well described. It was reported that post-OLT hyperuricaemia was related to immunosuppressants, primarily CNIs (CsA and FK506), which decrease UA excretion by reducing GFR and increasing UA reabsorption in renal tubules. The incidence of hyperuricaemia was much higher in recipients treated with CsA than in those treated with FK506 in some reports. Multiple mechanisms are related to renal damage caused by UA, including contributions to the development of hypertension [25], activation of the renin-angiotensin-aldosterone system (RAAS) [26–28], inflammation, and oxidative stress reactions [29]. In patients with CKD, a high level of serum UA was associated with cardiovascular diseases [30], a higher risk of incident RRT, and all-cause mortality [31]. It is important to reduce UA levels in OLT recipients to prevent the occurrence of CKD and improve prognosis. The treatments include changing the unhealthy lifestyle of patients and using drugs such as febuxostat, allopurinol, and topiroxostat. In addition, CNIs should be avoided, or the dose should be reduced as much as possible. Some reports have shown that the reduction or even withdrawal of CNIs after OLT combined with MMF or mTOR inhibitors can contribute to the reduction in UA levels [32, 33]. Therefore, serum UA levels should be closely monitored by clinicians during follow-up. Immunosuppression programs should be individualized according to the specific situation of the recipient. Due to its negative impact on prognosis, serum levels of uric acid were monitored and intervened carefully for all patients in our centre. The HR of UA at 3 months was low in this study, which might be related to the intervention of lowering UA during follow-up.

Another risk factor for CKD after OLT was low Hb levels at 3 months. The association between preoperative Hb and CKD after OLT was found in a previous Chinese cohort study [6]. In our study, low Hb at 3 months after OLT may contribute to CKD by reducing the oxygen capacity of the blood, enhancing oxidative stress, and impairing haemostasis. In addition, a recent study from Korea revealed that CKD patients with anaemia are at high risk for hyperuricaemia, which may further promote the development of CKD. The reason for the decrease in Hb at 3 months may be related to the use of immunosuppressive medications. MMF, azathioprine, and sirolimus are common causes of bone marrow suppression, which thus leads to a decrease in Hb after OLT [34].

It is well known that the dose-dependent nephrotoxicity of CNIs also plays an important role in the occurrence of new-onset CKD. In our study, the average plasma concentration of CsA at 3 months was an independent risk factor for the development of post-OLT CKD. Calcineurin stimulates vascular endothelial cells to secrete endothelin, release angiotensin II and overexpress transforming growth factor-β. This process is accompanied by a decrease in stromal degrading enzyme activity, leading to the hyperconstruction of glomerular arterioles, hyalinosis, chronic thromboembolism, and the excessive synthesis of extracellular matrix. Finally, it results in tubule atrophy and interstitial fibrosis and reduces renal blood flow and glomerular filtration [6]. However, the concentration of tacrolimus was not included in the nomogram model. Most of our recipients were on long-term immunosuppression with tacrolimus, and interestingly, CKD recipients had significantly lower levels of tacrolimus. This finding was consistent with the finding of some previous studies, which did not show a correlation between tacrolimus levels and the development of CKD [35]. This may be due to the adjustment of tacrolimus dose in patients with known renal impairment in our centre.

Finally, a nomogram model was constructed based on multivariable Cox regression analysis, which provides clinicians with a visual tool to understand the impact of predictors on renal function after OLT. In addition, by comprehensively analysing all predictors included, we can accurately calculate the probabilities of CKD for each recipient, making the results more personalized. After evaluation, the C-index of the nomogram model was 0.75 for the training set and 0.80 for the validation set, indicating that the nomogram model has a strong ability to distinguish patients with and without CKD. The calibration curves in the training set and validation set showed that the predicted probabilities of CKD at 1, 3, and 5 years after OLT were consistent with the observed probabilities, which indicates the accurate prediction ability of our model.

Several prediction models for CKD after OLT have been developed. A CKD prediction formula was developed from New York based on urinary neutrophil gelatinase-associated lipocalin (uNGAL) at 24 h after OLT as the most important risk factor. However, uNGAL-24 h is not routinely examined in other centres. Moreover, this prediction model has not been validated in terms of its calibration abilities [36]. Recently, a biomarker model predictive of renal outcomes after liver transplantation was constructed based on a large population from multiple centres. The levels of β2-microglobulin and CD40 antigen included in the prediction model are not routinely examined in other centres. Despite the high area under the curve (AUC), no calibration verification was performed [37]. The pocket guide to identifying patients at risk for CKD after liver transplantation, which includes hepatitis C virus (HCV) as one of the predictive factors, is a reliable prediction model for German patients. However, it does not apply to Chinese patients because HCV is more common in Western countries than in China [38]. Another model from Italy has been validated through AUC calculation and calibration; however, the sample size was relatively small, and there was no visual tool developed for the model [2]. In addition, none of these prediction models have been used in Chinese recipients.

Compared with these models, our model has the following advantages. First, it is the first nomogram model constructed based on OLT recipients for predicting CKD. Second, the predictors included in our model were readily available at surgery or routinely tested in follow-up, which is conducive to the popularization of the model. Third, our model had excellent discrimination and calibration abilities in the training set and validation set. It is a simple and reliable tool to distinguish recipients at high risk for CKD after OLT at 1, 3, and 5 years. In addition, this model can be used earlier at 3 months after OLT, assisting clinicians in adjusting immunosuppressive drugs in advance.

This study had several limitations. First, this study had a retrospective single-centre design, and the sample size was small, so the model should be evaluated prospectively in other large multi-centre studies to demonstrate its applicability. Second, the use of MDRD to assess renal function may lack accuracy in candidates awaiting liver transplantation, possibly because of reduced muscle mass and/or hyperbilirubinemia. It would overestimate renal function in patients with poor function (GFR < 40 mL/min) and underestimate renal function in patients with reasonable renal function (GFR > 40 mL/min) [17]. However, this is the most frequently used method to estimate kidney function. Third, due to data deficiency, we did not analyse the relationship between urinalysis data such as proteinuria and postoperative CKD. Fourth, to more accurately clarify the impact of CKD on the long-term prognosis after OLT, it is necessary to conduct a longer follow-up study.

In conclusion, the renal function of most recipients decreased and recovered rapidly in the first week after OLT and then decreased slowly year by year. Patients with severe CKD had a poor survival prognosis. We constructed a nomogram model for predicting post-OLT CKD for the first time. With excellent discrimination and calibration, this nomogram model can accurately predict patients at high risk for CKD after OLT. Therefore, we can take measures to prevent or slow the development and progression of CKD in advance, such as reducing UA levels, improving Hb levels, and withdrawing or minimizing the use of CNIs in follow-up.

## Data Availability

The datasets used and/or analyzed during the current study are available from the corresponding author on reasonable request.

## Abbreviations

AKI: Acute kidney injury
AST: Aspartate aminotransferase
ALT: Alanine transaminase
AIC: Akaike’s information criterion
BMI: Body mass index
BUN: Blood urea nitrogen
CKD: Chronic kidney disease
CKD-MDRD: the Modification of Diet in Renal Disease equation
CysC: Cystatin
C1q: Complement C1q
CsA: Cyclosporin A
CI: Confidence interval
DBIL: Serum direct bilirubin
eGFR: Estimated glomerular filtration rate
ESRD: End-stage renal disease
FK506: Tacrolimus
Glu: Glucose
HR: Hazard ratio
IQR: Interquartile range
IRI: Ischemia reperfusion injury
IBIL: Serum indirect bilirubin
KDIGO: Kidney Disease Improving Global Outcomes
LVEF: Left ventricular ejection fraction
LDH: Lactate dehydrogenase
MAP: Mean arterial pressure
MELD: Model for End-Stage Liver Disease
NLR: Neutrophil lymphocyte ratio
N/LP: Ratio of Neutrophil to lymphocyte and platelet
OLT: Orthotopic liver transplantation
POD: Postoperative day
Peak AST: AST peak value within first 24 h after OLT
PLR: Platelet lymphocyte ratio
RRT: Renal replacement therapy
sCr: Plasma creatinine
TBIL: Serum total bilirubin
UA: Uric acid
